# Social Disadvantage, Maternal Psychological Distress, and Difficulties in Children’s Social-Emotional Well-Being

**DOI:** 10.3390/bs8110103

**Published:** 2018-11-11

**Authors:** Robert J. Noonan, Stuart J. Fairclough

**Affiliations:** Department of Sport and Physical Activity, Edge Hill University, Ormskirk L39 4QP, UK; Stuart.fairclough@edgehill.ac.uk

**Keywords:** social-emotional well-being, maternal psychological distress, neighborhood deprivation, maternal education

## Abstract

This study used data from wave four of the United Kingdom (U.K.) Millennium Cohort Study to examine whether there is an individual (i.e., maternal education) and area-level social disadvantage (i.e., neighborhood deprivation) gradient to difficulties in social-emotional well-being (SEW) in 7-year-old English children. We then investigated to what extent maternal psychological distress (Kessler 6 score) explains the relationship between social disadvantage indicators and boys’ and girls’ SEW difficulties. Subjects consisted of 3661 child–mother dyads (1804 boys and 1857 girls). Results discerned gender differences in the effect social disadvantage indicators have on child SEW difficulties. Maternal education had a comparable effect on boys’ and girls’ SEW difficulties, but a steeper neighborhood deprivation gradient was evident for boys’ SEW difficulties compared to girls’ SEW difficulties. The effect of each social disadvantage indicator on boys’ and girls’ SEW difficulties was for most part direct and strong (*p* ≤ 0.001) rather than through maternal psychological distress, suggesting that the theoretical framework was incomplete. Here we demonstrate that where children are positioned on the social disadvantage gradient matters greatly to their SEW. Improving the living conditions and health of mothers with psychological distress may offer a pathway to improve child SEW.

## 1. Introduction

Social-emotional well-being (SEW) relates to a child’s self-perception and their ability to interact with others. Children that experience SEW difficulties often demonstrate negative emotions and behaviors, such as anxiety, depression, and hyperactivity, and struggle to maintain friendships with peers [1]. SEW difficulties in childhood can severely impact upon children’s academic achievement and mental health in adulthood, which both convey broad negative health and economic consequences throughout the life course [2]. The prevalence of child SEW difficulties increased between 1974 and 1999 in the United Kingdom (U.K.), and in 2004, 1 in 10 children were reported to have a diagnosable SEW difficulty [3]. Evidence shows that social disadvantage is a strong predictor of child SEW difficulties [4].

Traditionally, research in this area has studied the inverse effects of individual characteristics of social disadvantage, such as low family income or maternal education, on child SEW [5,6]. However, these indicators of social disadvantage do not capture the physical neighborhood environment within which socially disadvantaged children live (i.e., high social disorder, poor housing conditions, limited access to services and aesthetic neighborhood features) which can also predispose them to SEW difficulties [7,8,9]. As such, each social disadvantage indicator may influence the development of child’s SEW differently. Moreover, some evidence shows gender differences in SEW difficulties [10,11]; therefore, the influence of social disadvantage on child SEW could be gender-specific. However, to date no study has examined the influence of different indicators of social disadvantage on boys’ and girls’ SEW or determined whether a social gradient to SEW exists among boys and girls in the U.K.

The mechanisms linking social disadvantage and child SEW remain largely underexplored. Previous research in this area has predominantly been guided by the Family Investment Model (FIM) and Family Stress Model (FSM [12]). The FIM relates to the financial aspect of social disadvantage and postulates that low family income limits the purchasing of materials, experiences, and services to enhance child development and well-being [13]. The FSM rather addresses how social disadvantage influences child development and well-being through its effect on parental psychological distress and subsequent disruption of parent–child interactions/relations [14]. Previous studies investigating mechanisms underlying the association between social disadvantage and children’s SEW have been conducted mainly in the United States, which experiences greater income inequality compared to the U.K. [15,16]. Further, much of the evidence regarding SEW in U.K. children relates to younger children [5,13] who are less likely to demonstrate SEW difficulties compared to older children.

The study of child SEW is further complexed by social disadvantage and maternal psychological distress being mutually associated with child SEW, while not being independent of each other [17]. Indeed, extensive evidence shows that factors linked to social disadvantage, including low maternal income, precarious employment and housing insecurity, are strong predictors of maternal psychological distress [18]. It is important to recognize though that mothers experience particular combinations of these factors which are likely to carry varying levels of risk to their children. While the role of maternal psychological distress is considered important in the relationship between family income and early child SEW [13,19], it is unknown whether the relationship holds for older children, (boys and girls) and is consistent across different indicators of social disadvantage.

Therefore, to address these research gaps, the present study aimed to examine whether there is an individual (i.e., maternal education) and area-level social disadvantage (i.e., neighborhood deprivation) gradient to SEW in 7-year-old English children. A secondary aim was to investigate to what extent maternal psychological distress (Kessler 6 score) explains the relationship between different indicators of social disadvantage (i.e., maternal education and neighborhood deprivation) and boys’ and girls’ SEW. Firstly, it is hypothesized that children in the lowest maternal education group and those living in the most deprived neighborhoods would be at greatest risk of SEW difficulties, but the strength of these relationships would differ by gender and social disadvantage indicators. Secondly, it is hypothesized that maternal psychological distress would mediate the association between social disadvantage indicators and child SEW, and associations would vary by gender and social disadvantage indicators.

## 2. Materials and Methods

### 2.1. Participants

Data for this study were derived from wave four of the U.K. Millennium Cohort Study (MCS). The MCS is a nationally representative U.K. sample of children born between September 2000 and January 2002. The sample design allowed for over-representation of ethnic minority families and families living in areas of high neighborhood deprivation. The first survey was conducted throughout 2001–2002 when the children were aged 9 to 11 months old, and involved 18,819 children from 18,533 families. Subsequent surveys were administered at the ages of 3, 5 and 7 years. All measures were collected in the child’s home. To ensure consistency with earlier analyses [20], the present study only included English children who had complete anthropometric and physical activity data alongside the variables of interest, and for whom the main respondent to the study was the biological mother. Applying these criteria resulted in a final sample of 3661 (98.5%) child–mother dyads (1804 boys and 1857 girls). Ethical approval for the original study was granted by the Northern and Yorkshire Research Ethics Committee (07/MRE03/32).

### 2.2. Measures

#### 2.2.1. Social-Emotional Well-Being Difficulties

The Strengths and Difficulties Questionnaire (SDQ) is a validated questionnaire to assess child SEW (http://www.sdqinfo.com/). The SDQ has been used as a measure of SEW difficulties globally [21]. The SDQ comprises 25 items covering five domains of behavior including hyperactivity, emotional symptoms, conduct problems, peer problems and prosocial behavior. Each item has three response categories: ‘not true’, ‘somewhat true’, or ‘certainly true’. ‘Somewhat true’ is always scored as 1, but the scoring of ‘not true’ and ‘certainly true’ responses varies across items (0 or 2). Higher scores on the prosocial behavior subscale reflect strengths, whereas higher scores on the other four subscales reflect difficulties. An overall difficulties score is derived from the sum of all scales, excluding the prosocial behavior domain [21]. Overall scores ranged from 0 to 40, with higher scores denoting more difficulties (i.e., lower SEW). SDQ difficulties were defined as a score of ≥14.

#### 2.2.2. Maternal Psychological Distress

The Kessler 6 scale was used to assess maternal psychological distress [22]. Mothers reported how often over the previous 30 days they had felt everything was an effort, depressed, hopeless, restless or fidgety, worthless, and nervous. For each item, mothers indicated whether they had felt this way (1) never; (2) a little; (3) sometimes; (4) most of the time; or (5) all of the time. Responses range from 0 (none of the time) to 4 (all of the time) and are summed to produce an overall score ranging from 0–24. Serious psychological distress was defined as a score of ≥13.

#### 2.2.3. Social Disadvantage

Maternal education and neighborhood deprivation were used as indicators of social disadvantage. Mothers reported their highest educational qualification achieved by the time their child was 7 years of age. Responses were classified into four categories (Higher education/degree (1); A-levels (2); O levels/GCSE A*–C (3); CSEs/GCSE D–G/none (4)). Lower levels of qualifications represented higher social disadvantage. Neighborhood deprivation was calculated from home postcodes using the 2004 English Indices of Multiple Deprivation (IMD [23]). The IMD is a U.K. Government-produced measure comprising seven areas of deprivation (income, employment, health, education, housing, environment, and crime). Decile scores were collapsed into five categories ranging from the least to most deprived neighborhoods in England. Higher decile scores represented higher social disadvantage.

### 2.3. Data Analysis

Analyses were conducted using SPSS v. 24 (SPSS Inc; Chicago, IL, USA) and statistical significance was set at *p* ≤ 0.05. Descriptive statistics were calculated for all measured variables. Independent samples *t*-tests and chi-squared tests (χ²) were used to compare gender differences in continuous and categorical variables, respectively. Preliminary analyses revealed gender differences in SEW and confirmed that there was an interaction between indicators of social disadvantage (i.e., maternal education and area deprivation) and gender. Therefore, boys and girls were separated for all analyses. Gender-specific multinomial logistic regression analyses examined the likelihood of children being classified as having SEW difficulties based on maternal education and neighborhood deprivation level. The lowest level of maternal education and the highest level of neighborhood deprivation were chosen as the reference categories. For the main analyses, the outcome variable was total SDQ scores. Gender-specific mediation was assessed through regression analyses using the PROCESS macro for SPSS (http://www.processmacro.org/index.html [24]). Nonparametric bootstrapping analysis estimated direct and indirect effects in models [24]. Bootstrapping is a non-parametric resampling method that uses confidence intervals to estimate the size of indirect effects. The bootstrapping approach makes no assumption about the shape of the sampling distribution of the indirect effect and has been shown to enhance statistical power and Type I error control compared to other techniques [25]. Consistent with recommendations, 95% bias corrected, and accelerated confidence intervals were employed, and 5000 bootstrapping re-samples were run [25]. Mediation is evident when zero is not included within the lower and upper bound confidence intervals.

## 3. Results

Data were available for 3661 mother–child dyads (1857 girls). The descriptive characteristics of the sample and gender differences in measured variables are presented in Table 1. In this sample, 17% and 23% of children lived in the most and least deprived neighborhoods, respectively, 10% of children had a SEW difficulty, 14% of mothers had no qualification compared to 46% who had degree-level education, and 2% of mothers had psychological distress. With regards to gender differences, boys recorded higher SDQ scores than girls, and 12.3% of boys were classified as having a SEW difficulty compared to 8.5% of girls (*p* ≤ 0.001).

### 3.1. Research Aim 1

Multinomial logistic regression analyses revealed that boys living in the most deprived neighborhoods were more likely to have SEW difficulties compared with boys living in the fourth to least (odds ratio, OR = 1.78; *p* ≤ 0.01; Table 2), third to least (OR = 2.00; *p* ≤ 0.001), second to least (OR = 2.32; *p* ≤ 0.001) and least deprived neighborhoods (OR = 2.85; *p* ≤ 0.001). Girls living in the most deprived neighborhoods were more likely to have SEW difficulties compared with girls living in the least deprived neighborhoods (OR = 2.57; *p* ≤ 0.001).

Boys from the lowest maternal education group were more likely to have SEW difficulties compared with boys from the third to highest (OR = 2.47; *p* ≤ 0.001), second to highest (OR = 2.33; *p* ≤ 0.001), and highest maternal education group (OR = 3.49; *p* ≤ 0.001). A steep maternal education gradient to SEW difficulties was also observed among girls. Girls from the lowest maternal education group were more likely to have SEW difficulties compared with girls from the third to highest (OR = 2.05; *p* ≤ 0.01), second to highest (OR = 2.37; *p* ≤ 0.01), and highest maternal education group (OR = 3.51; *p* ≤ 0.001).

### 3.2. Research Aim 2

**Figure 1:**
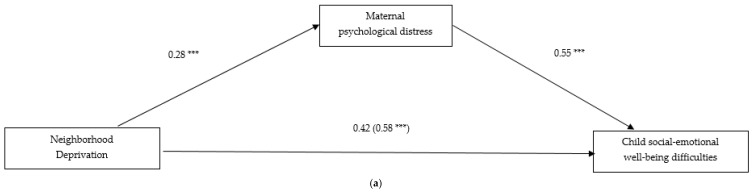

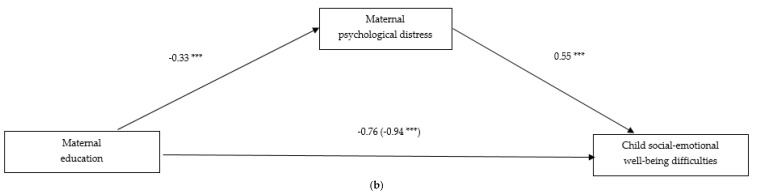

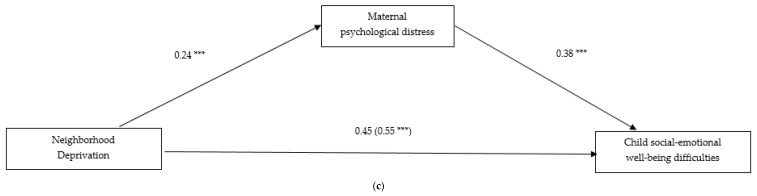

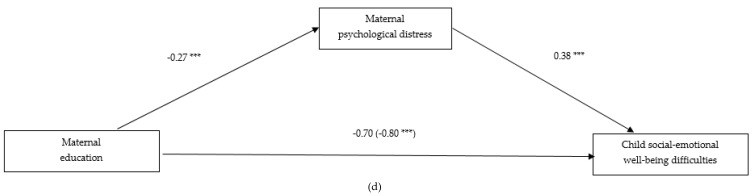
(**a**,**b**) Regression models predicting social-emotional well-being difficulties for boys. Values signify unstandardized regression coefficients. The direct effect of neighborhood deprivation and maternal education on social-emotional well-being are outside parentheses. The total effect is inside parentheses. *** *p* ≤ 0.001; (**c**,**d**) Regression models predicting social-emotional well-being difficulties for girls. Values signify unstandardized regression coefficients. The direct effect of neighborhood deprivation and maternal education on social-emotional well-being are outside parentheses. The total effect is inside parentheses. *** *p* ≤ 0.001.

Figure 1 displays unstandardized regression coefficients for the gender-specific mediation models. In both models, neighborhood deprivation and maternal education were the independent variables and total SDQ scores the dependent variable. Maternal education was the mediator (panel A and B). Results of the indirect effects for boys and girls are presented in Table 3. Figure 1 shows that neighborhood deprivation and maternal education were related to maternal psychological distress for boys (β = 0.28, *p* ≤ 0.0001; β = −0.33, *p* ≤ 0.0001) and girls (β = 0.24, *p* ≤ 0.0001; β = −0.27, *p* ≤ 0.0003). Maternal psychological distress was related to boys’ (β = 0.55, *p* ≤ 0.001), and girls’ SDQ scores (β = 0.38, *p* ≤ 0.0001).

In the neighborhood deprivation model for boys (Figure 1, panel A), the bootstrap procedure revealed that the total effect of neighborhood deprivation on SDQ scores was significant (β = 0.58, *p* ≤ 0.0001). When the mediator was included in the model, the direct effect of neighborhood deprivation on boys’ SDQ scores reduced but remained significant, suggesting partial mediation (β = 0.42, *p* ≤ 0.0001). Maternal psychological distress displayed an indirect effect, β = 0.15; 95% confidence interval (CI) = 0.09–0.22; *p* ≤ 0.0001 (Table 3). Therefore, the effect of neighborhood deprivation on boys’ SDQ scores was partially mediated by maternal psychological distress. In the maternal education model for boys (Figure 1, panel B), the bootstrap procedure revealed that the total effect of maternal education on SDQ scores was significant (β = −0.94, *p* ≤ 0.0001). When the mediator was included in the model, the direct effect of maternal education on boys’ SDQ scores reduced but remained significant, suggesting partial mediation (β = −0.76, *p* ≤ 0.0001). Maternal psychological distress displayed an indirect effect, β = −0.18; 95% CI = −0.27–−0.10; *p* ≤ 0.0001 (Table 3). Therefore, the effect of maternal education on boys’ SDQ scores was partially mediated by maternal psychological distress.

In the neighborhood deprivation model for girls (Figure 1, panel C), the bootstrap procedure revealed that the total effect of neighborhood deprivation on SDQ scores was significant (β = 0.55, *p* ≤ 0.0001). When the mediator was included in the model, the direct effect of neighborhood deprivation on girls’ SDQ scores reduced but remained significant, suggesting partial mediation (β = 0.45, *p* ≤ 0.0001). Maternal psychological distress displayed an indirect effect, β = 0.09; 95% CI = 0.05–0.14; *p* ≤ 0.0001 (Table 3). Therefore, the effect of neighborhood deprivation on girls’ SDQ scores was partially mediated by maternal psychological distress. In the maternal education model for girls (Figure 1, panel D), the bootstrap procedure revealed that the total effect of maternal education on SDQ scores was significant (β = −0.80, *p* ≤ 0.0001). When the mediator was included in the model, the direct effect of maternal education on girls’ SDQ scores reduced but remained significant, suggesting partial mediation (β = −0.70, *p* ≤ 0.0001). Maternal psychological distress displayed an indirect effect, β = −0.10; 95% CI = −0.17–−0.04; *p* = 0.0004 (Table 3). Therefore, the effect of maternal education on girls’ SDQ scores was partially mediated by maternal psychological distress.

## 4. Discussion

This study is the first to examine associations between individual and area-level indicators of social disadvantage and child SEW difficulties and investigate the mediating effect of maternal psychological distress on the social disadvantage–child SEW difficulties relationship. In the present study, both indicators of social disadvantage were strongly positively associated with child SEW difficulties, and further analyses revealed marked child SEW inequalities between the most and least socially disadvantaged categories. This finding supplements previous work which evidenced a steep social gradient to child obesity in England based on the same social disadvantage indicators used in the present study [20]. Together the findings underscore the importance of assessing child health across a range of social disadvantage indices. Failure to do so could potentially mask the pervasive effect of social disadvantage on child health. We also revealed that the strength of association between social disadvantage and child SEW difficulties varied by social disadvantage indicator. Indeed, aside from maternal education being a stronger predictor of boys’ and girls’ SEW difficulties compared to neighborhood deprivation, a steeper gradient to child SEW difficulties was also evident for maternal education relative to neighborhood deprivation. This finding could be due to maternal education being a stronger indicator of low income and financial hardship, which are central facets of the FIM [12]. Low maternal education has been found to be a strong predictor of child SEW difficulties in previous U.K. [26] and European research [27]. However, here we draw specific attention to the social gradient and child SEW. Where children are positioned on the social disadvantage gradient matters greatly to their SEW.

A second study objective was to examine the mediating effect of maternal psychological distress on the relationship different indicators of social disadvantage and child SEW difficulties. Our hypothesis was that the increased risk of child SEW difficulties may be explained by maternal psychological distress. This hypothesis was partially supported and corroborates somewhat with the FSM [12]. However, the effect of each social disadvantage indicator on child SEW difficulties was for most part direct and strong rather than through maternal psychological distress, suggesting that the theoretical framework was incomplete. While no previous studies have examined the exact mediation mechanisms that were the focus of the present study, the results from our study are consistent with younger samples. For example, previous U.K. research involving young children revealed that the social disadvantage–child SEW difficulties relationship remained significant after accounting for parental stress and other factors known to influence child behavior [19]. Together, these findings indicate that additional research is needed to explain the processes by which different indicators of social disadvantage affect child SEW difficulties. Future research should investigate the mediating effects of child stress and lifestyle factors (e.g., dietary intake) and/or neighborhood attributes on the social disadvantage–child SEW difficulties relationship.

A novel element of this study was the examination of gender-specific relationships between social disadvantage, maternal psychological distress and child SEW difficulties. The gender-specific multinomial logistic regression analyses discerned gender differences in the effect social disadvantage indicators have on child SEW difficulties. Interestingly, while maternal education had a comparable effect on boys’ and girls’ SEW difficulties, a steeper neighborhood deprivation gradient was evident for boys’ SEW difficulties compared to girls’ SEW difficulties. This finding would suggest that position on the deprivation gradient matters more for boys’ SEW compared to girls’ SEW. Moreover, in both mediation models, larger total and indirect effects were observed for boys compared to girls. Together, these findings propose that the independent and combined effect of social disadvantage and maternal psychological distress predispose boys to SEW difficulties more so than girls. Presently, it is largely unknown whether differential effects exist for neighborhood and child SEW relationships. Quasi-experimental studies such as the Moving to Opportunity program gave residents living in some of America’s most socially disadvantaged neighborhoods the opportunity to move to more affluent neighborhoods [28]. The program resulted in reduced rates of conduct disorder among girls but increased rates of depression and conduct disorder among boys. These findings suggest that gendered intervention programs are needed and that boys may need additional program support than girls.

It is difficult to fully compare our findings to earlier work as these studies involved younger samples and did not explore gender differences [5,13,19]. However, a review study reported that maternal depression was more strongly associated with internalizing problems (e.g., emotional and peer symptoms) in girls than boys [29]. Here we assessed the influence of maternal psychological distress on total SDQ scores rather than specific internalizing and externalizing problems which may have contributed to the heterogeneity in results. There are a multitude of adversities associated with social disadvantage that collectively contribute to its pervasive impact on child SEW, and specifically, internalizing and externalizing problems [7,30]. A majority of interventions to improve child SEW have targeted only one social disadvantage risk factor (e.g., family income, neighborhood) and efforts targeting a range of social disadvantage risk factors are likely to be needed to bring about greater and more continued change [16]. However, additional research is needed to understand how the various individual, family, and neighborhood risk factors interact to create unconducive environments for boys’ and girls’ development and well-being.

Consistent with prior research this study revealed that maternal psychological distress was strongly influenced by social disadvantage [31], especially maternal education level, and strongly predicted both boys’ and girls’ SEW difficulties. Education level influences a mother’s employment opportunities which in part determines their income level. Although all mothers are likely to experience some level of stress, mothers with less education, income, and social status have fewer resources to mitigate that stress in comparison to mothers with higher education, income, and social status. Maternal psychological distress makes optimal parenting more challenging and has been shown to impact negatively on child well-being [17,29]. Compared to low education mothers, mothers with a high level of education are more likely to be cognizant of normative child development milestones as well as approaches appropriate to fulfilling the social-emotional and cognitive needs of their children as they develop [32]. Moreover, it is likely that highly educated mothers are more effective in providing the social support required to cope with the negative effect maternal psychological distress has on their child’s SEW [33]. Mother’s educational level may also directly influence father parenting behavior and father-child relations, given that a mother’s educational level influences their attribution of children’s behavior which in turn affects the quality of their parenting decisions and behavior [32,34]. Children of low-educated mothers experiencing maternal psychological distress are at the greatest risk of SEW difficulties and thus represent an important target group for future child SEW interventions.

Overall, the present study demonstrates the need for a broader recognition of maternal mental health as a foundation for children’s well-being. In this study, mothers with the lowest education and those living in the most deprived neighborhoods were most likely to report psychological distress and child SEW difficulties. Together these results suggest that the poorer well-being of mothers experiencing psychological distress and their children can potentially be attributed to their poorer living conditions, rather than mothers’ well-being per se. The results are consistent with a socio-ecological approach to child- and maternal health promotion [35], whereby changes to context can improve multiple family health outcomes [36]. Traditionally, U.K. policy efforts to reduce social inequalities in child health have dedicated resources on children themselves. The results of this study suggest that a more family-centered approach to child health promotion whereby efforts are focused on enhancing mothers’ mental health may benefit children’s SEW.

There are some study limitations to acknowledge. Firstly, maternal reported SDQ scores may have been subject to measurement error and social desirability bias and may reflect gendered stereotypes of children’s behavior. Furthermore, we did not include all key pathways of the FSM (i.e., parenting practices) which may also influence the social disadvantage–child SEW relationship. Moreover, the study design was cross-sectional and does not determine causality. It is also important to note that the results of this study are based on data collected in 2007–2008 and may underestimate the current magnitude of health inequalities among English children and their mothers. For example, since 2010 a range of Conservative Government-led austerity measures (including cuts to children and family support services, welfare benefits, and tax credits) have been implemented, which have in most part hit the poorest areas of England hardest [37]. Coupled with these policy changes, the U.K. has experienced a rapid rise in precarious employment [38] and housing insecurity [39], all of which are strong risk factors for adult psychological distress [40,41]. It is not unreasonable to assume that these societal changes will have impacted most on the living conditions and consequent health of children and families living in the most deprived areas of England, and in turn widened health inequalities.

## 5. Conclusions

Our study evidences marked social disadvantage inequalities in English children’s SEW at age 7 years. The results of this study reinforce strong associations between individual and area-level indicators of social disadvantage and child SEW difficulties. They show that where children are positioned on the social disadvantage gradient matters greatly to their SEW. We also demonstrate the influence maternal psychological distress has on the social disadvantage–child SEW difficulties relationship. Improving the living conditions and health of mothers with psychological distress may offer a pathway to improve child SEW.

## Figures and Tables

**Table 1:** Descriptive characteristics of full sample and gender differences.

Variables	All (*n* = 3661) Mean (SD)	Boy (*n* = 1804) Mean (SD)	Girl (*n* = 1857) Mean (SD)
Age (years)	7.22 (0.25)	7.22 (0.25)	7.22 (0.25)
Indices of Multiple Deprivation decile (%)			
Least deprived	23.10	23.20	23.00
Second	20.70	20.40	21.10
Third	20.70	20.70	20.70
Fourth	18.10	18.20	18.00
Most deprived	17.30	17.50	17.20
Maternal education level (%)			
None	26.60	25.30	27.90
GCSE	14.30	14.70	13.90
A-level	45.50	46.00	45.10
Degree	13.50	14.00	13.10
Strengths and Difficulties Questionnaire score	6.82 (4.95)	7.46 (5.18)	6.19 (4.64) *
Social-emotional well-being difficulty (%)	10.40	12.30	8.50 *
Kessler 6 score	2.84 (3.48)	2.86 (3.39)	2.81 (3.57)
Serious psychological distress (%)	2.40	2.10	2.70

Social-emotional well-being difficulty = ≥14 on the Strengths and Difficulties Questionnaire; Serious psychological distress = ≥13 on the Kessler 6 scale; * *p* ≤ 0.001.

**Table 2:** Gender-specific multinomial logistic regression associations between social disadvantage indicators and social-emotional well-being difficulties (*n* = 3661).

Variables	OR	95% CI	*p*
Boys			
Neighborhood deprivation			
Most deprived—Reference (*n* = 315)			
Fourth (*n* = 329)	1.78	1.16–2.71	≤0.01
Third (*n* = 374)	2.00	1.32–3.04	≤0.001
Second (*n* = 367)	2.32	1.50–3.59	≤0.001
Least deprived (*n* = 419)	2.85	1.84–4.43	≤0.001
Maternal education			
CSEs/GCSE D–G/None—Reference (*n* = 253)			
O-levels/GCSE A*–C (*n* = 456)	2.47	1.65–3.69	≤0.001
A-level (*n* = 265)	2.33	1.47–3.70	≤0.001
Degree (*n* = 830)	3.49	2.40–5.07	≤0.001
Girls			
Neighborhood deprivation			
Most deprived—Reference (*n* = 319)			
Fourth (*n* = 334)	1.02	0.62–1.68	0.94
Third (*n* = 384)	0.97	0.60–1.57	0.91
Second (*n* = 392)	1.55	0.92–2.62	0.10
Least deprived (*n* = 428)	2.57	1.44–4.59	≤0.001
Maternal education			
CSEs/GCSE D-G/None—Reference (*n* = 243)			
O-levels/GCSE A*–C (*n* = 518)	2.05	1.31–3.20	≤0.01
A-level (*n* = 259)	2.37	1.36–4.13	≤0.01
Degree (*n* = 837)	3.51	2.25–5.48	≤0.001

CI, confidence interval; OR, odds ratio; Social-emotional well-being difficulties = score of ≥14 on the Strengths and Difficulties Questionnaire.

**Table 3:** Indirect effects of neighborhood deprivation and maternal education on child social-emotional well-being difficulties through maternal psychological distress by gender (*n* = 3661).

				Normal Theory Tests		
	Bootstrap Effect	95% CI	Normal Effect	SE	z	*p*
Boys						
Neighbourhood deprivation—Social-emotional well-being difficulties						
Total effect	0.58	0.41–0.74				
Maternal psychological distress	0.15	0.09–0.22	0.15	0.03	4.72	<0.0001
Maternal education—Social-emotional well-being difficulties						
Total effect	−0.94	−1.15–−0.73				
Maternal psychological distress	−0.18	−0.27–−0.10	–0.18	0.04	–4.48	<0.0001
						
Girls						
Neighbourhood deprivation—Social-emotional well-being difficulties						
Total effect	0.55	0.40–0.69				
Maternal psychological distress	0.09	0.05–0.14	0.09	0.02	3.97	0.0001
Maternal education—Social-emotional well-being difficulties						
Total effect	−0.80	−0.99–−0.62				
Maternal psychological distress	−0.10	−0.17–−0.04	−0.10	0.03	−3.51	0.0004

Note. Bootstrap-generated confidence intervals. CI = confidence interval; SE, standard error.
